# CABS-fold: server for the *de novo* and consensus-based prediction of protein structure

**DOI:** 10.1093/nar/gkt462

**Published:** 2013-06-08

**Authors:** Maciej Blaszczyk, Michal Jamroz, Sebastian Kmiecik, Andrzej Kolinski

**Affiliations:** Laboratory of Theory of Biopolymers, Faculty of Chemistry, University of Warsaw, Pasteura 1, 02-093 Warsaw, Poland

## Abstract

The CABS-fold web server provides tools for protein structure prediction from sequence only (*de novo* modeling) and also using alternative templates (consensus modeling). The web server is based on the CABS modeling procedures ranked in previous Critical Assessment of techniques for protein Structure Prediction competitions as one of the leading approaches for *de novo* and template-based modeling. Except for template data, fragmentary distance restraints can also be incorporated into the modeling process. The web server output is a coarse-grained trajectory of generated conformations, its Jmol representation and predicted models in all-atom resolution (together with accompanying analysis). CABS-fold can be freely accessed at http://biocomp.chem.uw.edu.pl/CABSfold.

## INTRODUCTION

The knowledge of 3D protein structures is important for understanding the molecular mechanisms of life. Owing to the enormous development of experimental studies, ∼90 000 protein structures are now known. However, they still represent a small fraction of all sequences available.

There are two kinds of qualitatively different classes of approaches to structure modeling. The first one is comparative modeling. With an increasing number of known protein structures for a large fraction of new proteins, it is possible to find realistic sequence alignments with structural templates and use these alignments in model building. On the other hand, there are *de novo* methods, involving molecular simulation of protein folding processes (1,2) or fragment assembly (3,4). In this context, the present CABS-fold server is unique: it enables a purely *de novo* structure assembly or uses the same CABS (C-Alpha, c-Beta, Side-chain) algorithm with a variety of distance restraints. These restraints could be derived from sequence alignments with proteins of known structures and/or from fragmentary (or complete) experimental data of various accuracies. Restraints could be applied to the entire sequence studied or to its fragments and with different strength levels.

The CABS algorithm is one of the most efficient tools for protein structure prediction. Research groups employing CABS-based methods have scored well in Critical Assessment of techniques for protein Structure Prediction (CASP) or have demonstrated accurate structure predictions for difficult *de novo* (or unclearly homological) targets (5–7).

The CABS model is based on a coarse-grained representation of protein chains and uses statistical potentials derived from known protein structures. The Monte Carlo sampling procedure uses randomly generated small local modifications of the model chains. The long sequence of such steps provides realistic picture of long-term dynamics (1,8–11). Obviously, owing to the discretization of the conformational space, short-time dynamics is poorly defined, and, consequently, the timescale of long-term CABS dynamics needs to be fitted to the long-time dynamics of atomistic models and/or experimental measurements of dynamic properties. It should also be pointed out that in contrast to most of other structure prediction algorithms, CABS is suitable for both: *de novo* and comparative modeling tasks. The level of resolution of the coarse-grained CABS protein structure representation seems to be optimal for fast Monte Carlo simulations and realistic reconstruction of all-atom models (9,12). This also makes CABS unique compared with other freely accessible protein modeling tools.

## CABS-fold METHOD

**Figure 1.**
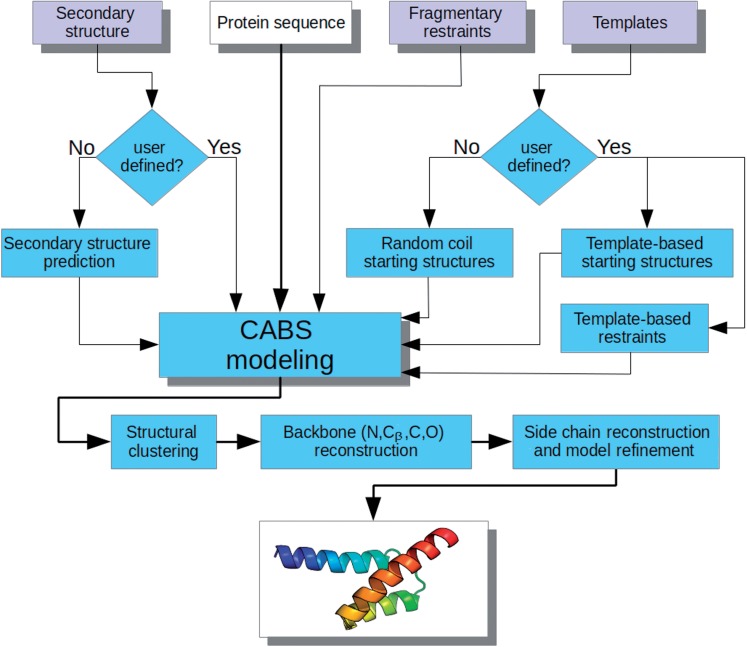
CABS-fold pipeline.

The CABS-fold server uses a unified multiscale modeling pipeline, which merges efficient exploration of the conformational space by the coarse-grained CABS model (13) with the atomic-level fine-tuning of the predicted models (see Figure 1). CABS-fold sampling is controlled by a Replica Exchange Monte Carlo scheme with 20 replicas spanning the specified temperature range. For user convenience, two simulation modes are pre-defined: *de novo* modeling (template-free) and consensus modeling (based on structural templates). In both simulation modes, user-defined distance restraints and a secondary structure can be applied.

As shown in Figure 1, the starting structures are either template(s)-based (the missing template fragments are automatically connected with a coil-type structure) or entirely random coil conformations (*de novo* modeling mode). The templates are also the basis for the generation of distance restraints. The restraints are defined as a list of pairwise distance ranges between the *i*-th and the *j*-th Cα atoms (during simulation, an energy penalty is applied if the position of the restrained Cα atoms deviates from the given range).

The way of setting up the pairwise distance ranges depends on template similarity. First, to assess template similarity, GDT_TS (Global Distance Test - Total Score) values between all template pairs are calculated. If the minimum GDT_TS between the templates is ≥0.3, the restraints for Cα atoms at *i*-th and *j*-th positions are defined as:

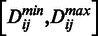

where 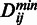 is the minimum observed distance between Cα atoms at positions *i*-th and *j*-th observed in a set of templates (analogically, 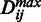 is the maximum observed distance).

If the set of templates contains at least one pair showing significant dissimilarity (GDT_TS < 0.3), distribution of the distances is used to define the restraints:

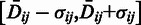

where 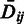 is the mean of observed distances between Cα atoms at *i*-th and *j*-th positions in a set of templates and 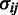 is standard deviation (see also the Input data paragraph).

A specific case of the consensus modeling mode is when a single template is provided. If so, the applied distance restraints enforce template structure on the template-covered protein fragments, whereas the remaining template-free fragments are modeled in a *de novo* fashion.

The resulting trajectories from the CABS algorithm are clustered using the K-means method. For each cluster, one representative model, either an average cluster structure or a cluster medoid (the model that averages dissimilarity to all models in a cluster is minimal) is selected. The choice of the representative model depends on the average cluster RMSD (root mean square deviation) of the densest cluster. If its average RMSD is >2.15 Å, the medoid is chosen; otherwise, the average cluster structure is selected. Such a criterion is the result of our tests, which showed that in most cases the choice of the average structure leads to more accurate models (in terms of RMSD or GDT_TS). On the other hand, for clusters containing diverse models, the average structure can be unphysical (owing to the averaging of the atom positions). Cluster representatives are ranked according to cluster density (the first one is the densest).

The next step is models reconstruction and optimization using ‘Backbone Building from Quadrilaterals’ method (reconstruction of backbone atoms from the Cα trace) (14) and subsequently the ModRefiner method (15) (side chain reconstruction and final model optimization). ModRefiner has been tested on a large set of protein models constructed from both *de novo* and template-based structure predictions (15). The tests showed that in comparison with other state-of-the art reconstruction and optimization procedures, ModRefiner shows improvements in both global and local structures, which have more accurate side-chain positions, better hydrogen-bonding networks and fewer atomic overlaps.

### Method validation

**Figure 2.**
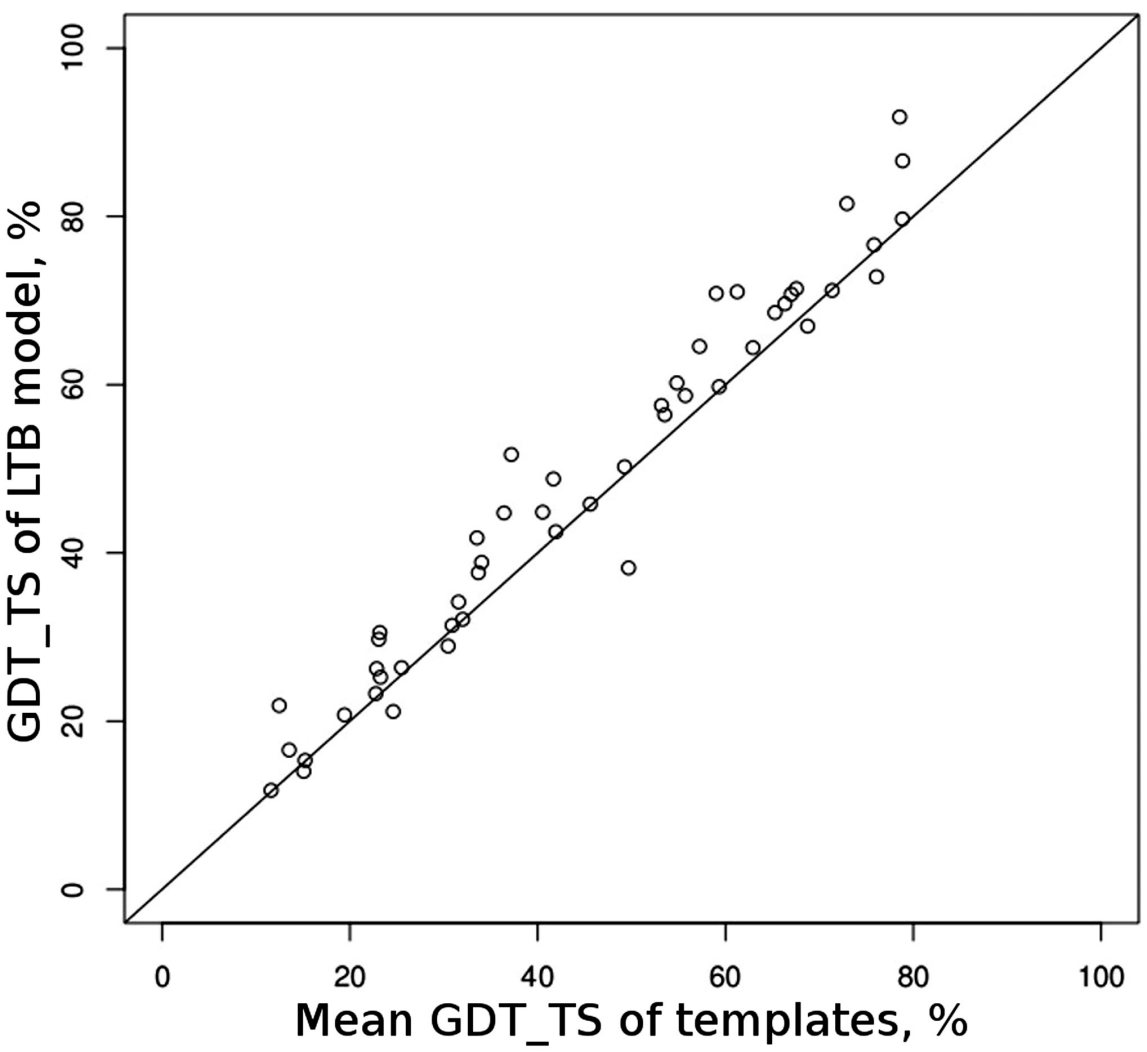
CASP9 results. Comparison of the accuracy of resulting models with the templates, measured by GDT_TS.

The consensus modeling protocol applied in CABS-fold was initially tested and optimized on a set of template-based targets from CASP8. Final tests were conducted in a blind CASP9 prediction experiment by the ‘LTB’ group (group no. 400). The purpose of the LTB group was to test the consensus modeling protocol on all CASP9 targets (thus on all difficulty levels) using prediction results from the chosen (potentially most reliable) automated servers as templates. The comparison of the quality of the resulting models and templates showed that in the great majority of cases, GDT_TS of the model was higher than the mean GDT_TS of the input structures (see Figure 2). Models of five domains (T0586-D1, T0628-D1, T0540-D1, T0594-D1, T0622-D1) provided by LTB were the best among all predictions submitted to CASP as the first models (only one group, PRMLS, no. 065, provided six of the best models, i.e. more). These most successful prediction results were generated from mostly consistent templates, for which the method was able to produce better prediction results than any of the templates used.

The performance of the CABS model in *de novo* loop modeling (in practice, this is the case when a single template with missing fragments is provided in the consensus modeling mode) was validated and compared with two classical modeling tools (16): MODELLER (17) and ROSETTA (4). Loops of various lengths, from 4 to 25 residues, were modeled assuming ideal target-template alignment of the remaining portions of the protein. It was shown that classical modeling with MODELLER was on average better for short loops, whereas CABS modeling was more effective for longer missing fragments.

In an application to the *de novo* modeling of large protein fragments or entire proteins (with or without fuzzy restraints), the CABS model was validated during CASP competitions as one of the leading approaches (5–7), as well as in purely *de novo* protein dynamics studies (1,9–11) demonstrating the ability of the algorithm to consistently fold small proteins from a highly denatured ensemble to a native-like ensemble (∼2 Å to the native), in a short central processing unit time (typically 2–3 h for short peptides and 8–12 h for small or large proteins).

### Input data

The required input data are the protein sequence in plain text or the FASTA format. Additionally, for a template-based mode, at least one or more template structures should be provided in a PDB format (or zip-compressed PDB files). Cα atom coordinates are required only; however, the server also accepts data containing backbone and/or side-chain atoms. Residue numbering should correspond to the query sequence order. Thus, the template(s) have to be already aligned with the query sequence. The accuracy of the final model critically depends on alignment accuracy (18). Certain well-established threading servers, such as HHPred (19) or SAM_T08 (20), or meta-servers, such as GeneSilico (21) or Pcons.net (22), can be used as a source of templates.

The maximum sequence length is 900 AA (the recommended sequence input length for *de novo* modeling is up to ∼120 residues).

Regardless of the chosen modeling mode, the user can provide information about the predicted secondary structure [if not, the PSI-PRED method (23) is automatically used]. The secondary structure should be defined for each residue in a three letter code: H, helix; E, extended state (beta sheet); and C, coil (less regular structures). Overpredictions of the regular secondary structure (H or E) are more dangerous for the quality of the results than underpredictions [i.e. for residues with an ambiguous secondary structure prediction assignment, it is better to assign coil (C) than a regular (H or E) structure].

In the advanced options (accessible under the ‘Advanced options’ link on the ‘Submit new job’ page), distance restraints can be added or removed. For example, entering a string ‘D 55,58,120–160’ deletes all restraints (automatically generated from templates) for residues 55, 58 and residues in the range of 120–160. It is also possible to add new restraints, e.g. a string ‘A 3 14 7.8 10.12’ defines the distance constraint between the 3rd and the 14th residue as the range of 7.8–10.12 Å.

The ‘Advanced options’ also enable setting up the temperature range (in the CABS simulation algorithm, temperature is the parameter that controls the acceptance ratio for new conformations using the Replica Exchange Monte Carlo scheme). The default temperature ranges are given in the ‘Advanced options’. For instance, the recommended range for *de novo* modeling is 3.5 − 1.0, for consensus modeling: 2.0 − 1.0. In a temperature of 3.5, most energetically unfavorable conformational changes are accepted; thus, the protein chain changes its conformation, quickly adopting mostly random coil structure, whereas in a temperature of 1.0, the chain is nearly frozen. Therefore, lowering the first temperature value limits potential structural rearrangements with respect to the starting structure. Setting up a flat temperature range, e.g. 2.0–2.0, enables the investigation of transient (less stable) folding conformers from the resulted trajectory, as shown in our protein mechanism studies (1,8–11).

### Output data

**Figure 3.**
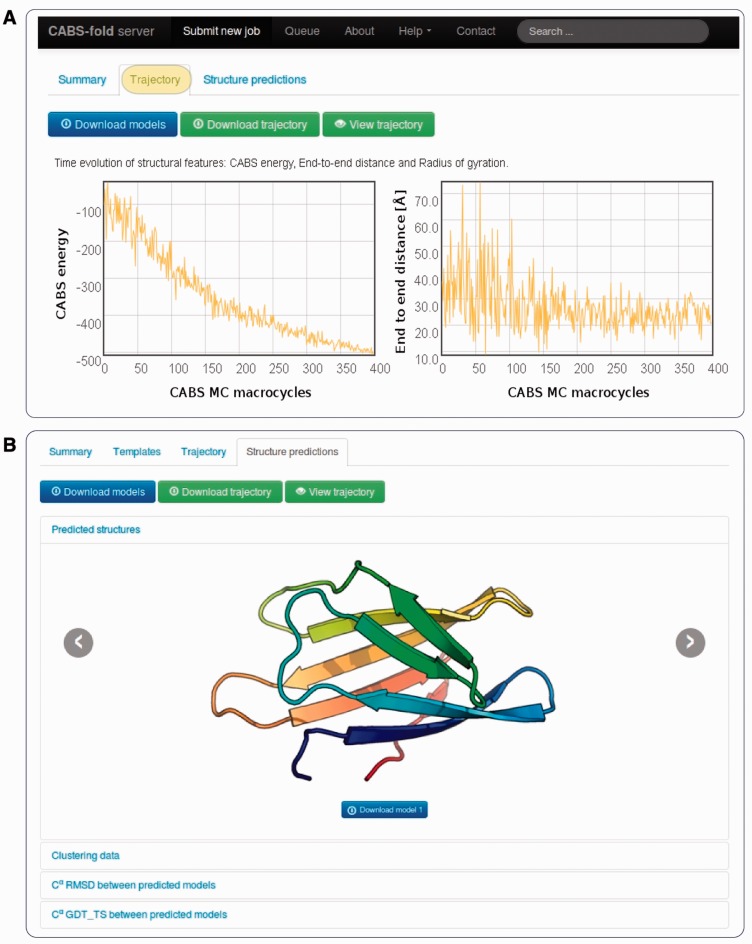
Example result pages: (**A**) Trajectory tab with the temporal evolution of structural features: CABS energy, end-to-end distance and radius of gyration (not shown, accessible from the bottom of the page) (**B**) Structure prediction tab presenting predicted models and cross-analysis of the models (in terms of RMSD and GDT_TS measures, accessible from the bottom of the page).

After computation is completed, the results are accessible from the unique job link and displayed under ‘Trajectory’ and ‘Structure Prediction’ tabs (see Figure 3). The ‘Trajectory’ tab shows changes in CABS energy, the radius of gyration and the end-to-end distance for the lowest energy replica snapshots (these results are also displayed ‘on-the-fly’ during computation). The trajectory in Cα representation can be downloaded as a PDB file and/or viewed online through the Jmol applet (significant conformational and/or rotational changes correspond to replica exchange events).

The resulting full atomistic models are visualized in the form of a slideshow and prepared for download in the ‘Structure prediction’ tab. The tab also presents the details of structural clustering and comparison analysis of the predicted models (under appropriate subtabs at the bottom of the page, see Figure 3).

### Documentation

The documentation of CABS-fold is available online and it can be accessed through links in the menu, at the top of every server page. Additionally, the web interface provides tooltips for download buttons or output graphics (to display a tooltip, drag the cursor over a picture or a particular button). The online documentation is updated on a regular basis according to user needs or method improvements.

### Availability

The CABS-fold server is free and open to all users and there is no login requirement. After clicking the Submit button (preceded by completing the input form), a web link to the results is provided, which the user can bookmark and access at a later time. Web links to the submitted jobs are displayed on a queue page, unless an option ‘Do not show my job on the queue page’ (available from the Submit page) is on.

## SERVER ARCHITECTURE AND PERFORMANCE

The CABS-fold server is equipped with a web interface written in HTML + PHP, which provides a convenient framework for pipeline control and presentation of output data. At the time of submission, a PHP script validates the correctness of data provided by the user and reports possible errors (wrong file formats, improper symbols in protein sequences, etc.). If all data are correct, the job is added to a MySQL database (‘pending’ status). A cron daemon script checks new records in the database and, if any exist, sends the job to the queue (‘in_queue’ status). If the server has free resources, the job starts (‘running’ status) invoking bash and python scripts using necessary data. As soon as the CABS-fold pipeline is complete, the job status is set to ‘done’. The job status, progress bar (showing approximate job progress) and simulation parameters are constantly displayed under a unique job link.

The server is equipped with a standard SGE 6.1 queue manager with the maximum number of parallel running tasks set to eight (out of 12). Every 2 min, the daemon checks if there are any new tasks, and, if they exist, it adds them to the queue manager. The runtime of a simulation depends mostly on the protein chain length and varies between 2 and 12 h (the approximate end time is given under the job link).

## FUNDING

Foundation for Polish Science MPD programme and TEAM project [TEAM/2011-7/6], co-financed by the EU European Regional Development Fund operated within the Innovative Economy Operational Program, from Polish National Science Centre [NN301071140] and Polish Ministry of Science and Higher Education [IP2011 024371]. Funding for open access charge: TEAM project [TEAM/2011-7/6], Foundation for Polish Science.

*Conflict of interest statement.* None declared.
